# Improving Kinematic Accuracy of Soft Wearable Data Gloves by Optimizing Sensor Locations

**DOI:** 10.3390/s16060766

**Published:** 2016-05-26

**Authors:** Dong Hyun Kim, Sang Wook Lee, Hyung-Soon Park

**Affiliations:** 1 Mechanical Engineering Department, Korea Advanced Institute of Science and Technology, Daejeon 34141, Korea; bomdon@kaist.ac.kr (D.H.K.); hyungspark@kaist.ac.kr (H.-S.P.); 2 Department of Biomedical Engineering, Catholic University of America, Washington, DC 20064, USA; leeb@cua.edu; 3 Center for Applied Biomechanics and Rehabilitation Research, MedStar National Rehabilitation Hospital, Washington, DC 20010, USA

**Keywords:** wearable hand device, sensor locations, kinematic accuracy, 3D measurement, thumb-joint angle estimation

## Abstract

Bending sensors enable compact, wearable designs when used for measuring hand configurations in data gloves. While existing data gloves can accurately measure angular displacement of the finger and distal thumb joints, accurate measurement of thumb carpometacarpal (CMC) joint movements remains challenging due to crosstalk between the multi-sensor outputs required to measure the degrees of freedom (DOF). To properly measure CMC-joint configurations, sensor locations that minimize sensor crosstalk must be identified. This paper presents a novel approach to identifying optimal sensor locations. Three-dimensional hand surface data from ten subjects was collected in multiple thumb postures with varied CMC-joint flexion and abduction angles. For each posture, scanned CMC-joint contours were used to estimate CMC-joint flexion and abduction angles by varying the positions and orientations of two bending sensors. Optimal sensor locations were estimated by the least squares method, which minimized the difference between the true CMC-joint angles and the joint angle estimates. Finally, the resultant optimal sensor locations were experimentally validated. Placing sensors at the optimal locations, CMC-joint angle measurement accuracies improved (flexion, 2.8° ± 1.9°; abduction, 1.9° ± 1.2°). The proposed method for improving the accuracy of the sensing system can be extended to other types of soft wearable measurement devices.

## 1. Introduction

In many clinical applications, accurate measurements of hand movements and/or postures are required [1,2]. A device that can accurately measure different hand postures would be valuable as it enables accurate and precise clinical assessment of hand function and dexterity. While, in clinical settings, finger- and thumb-joint ranges of motion (ROM) are typically measured using manual goniometers, goniometers are limited to static measurements and typically require a long period of time to collect. Measurement accuracy is also limited as it involves subjective inspection made by clinicians/therapists. Thus, a sensing system that allows automatic measurements of hand configuration could significantly improve clinical practice, as it will allow swift measurement of hand postures during static and/or dynamic tasks. Furthermore, an accurate sensing system could improve the performance of hand assistive devices. Recent hand rehabilitation devices enabled compact design through the use of remotely located actuators, which also improve usability and convenience [3,4,5]. However, many of these devices employ relatively simple open-loop control, as they do not feature sensing systems that can accurately measure joint angles [3,4,5]. Development of a hand configuration sensing system would enable monitoring and feedback-control of individual joint angles, which could help subjects achieve natural hand postures during various functional tasks. In addition to the clinical applications, it can be used as a precise sensing system for training skillful hand actions.

Among the 25 degrees of freedom (DOF) of hand kinematics [6], accurate measurement of thumb kinematics is especially important due to its position and unique functions. Unlike the fingers, opposition of the thumb can enable various grasp tasks such as power, pinch, and lateral grasp. However, accurate measurement of thumb kinematics is challenging due to the large number of DOFs associated with thumb movements, which are enabled, in large part, by the saddle-shaped trapezium located in the carpometacarpal (CMC) joint [7]. Furthermore, definition of the CMC-joint coordinate system is not unique [7,8,9,10,11], which makes conducting consistent and comparative analyses difficult. Despite the development of many compact hand sensing devices, there are few reports on the accuracy of thumb-joint-angle measurements [12,13,14,15,16]. Previous literature has cited the mean error of estimating the CMC-joint angle using the VPL DataGlove (VPL Research Inc., San Francisco, CA) as 11° ± 9° [15], 23° ± 7° for the CMC abduction angle and 11° ± 2° for the CMC rotation angle using the CyberGlove^®^ model (CyberGlove Systems LLC., San Jose, CA) CG1801 [16]. In clinical assessment, a maximum of 5° estimation error is considered acceptable [17], and these previously reported errors do not meet this requirement.

We postulate that there are two major issues that need to be addressed in order to improve results in CMC-joint angle estimation. First, previously reported hand configuration monitoring systems may have been limited by the use of kinematic models for the CMC-joint coordinate system that did not adequately account for the type(s) of sensor used (*i.e.*, bending sensors). In addition, unlike with bending sensors that can measure single-DOF-movements of the distal finger and thumb joints (e.g., distal interphalangeal and proximal interphalangeal joints), there can be a significant amount of crosstalk between flexible bending sensors used to measure two-DOF-movements of the CMC-joint. Significant crosstalk between multiple sensors attached to the dorsal surface of the hand can degrade the accuracy of measured multi-DOF movements.

Therefore, in this study, we propose a systematic approach to improving measurement accuracy of CMC-joint movements. First, we developed a new kinematic model of the CMC-joint suitable for the use of flexible bending sensors. Based on this model, using 3D images containing the topology of the skin surface near the CMC-joint, we developed a novel method for estimating the optimal location (position and orientation) of two bending sensors that minimizes crosstalk between the sensor outputs. Finally, we experimentally evaluated the accuracy of the proposed sensing system.

## 2. Kinematic Model of the Thumb

### 2.1. Anatomy and Kinematics of the CMC-Joint

The complex multi-DOF movements of the CMC-joint originate from the movement of the first metacarpal bone with respect to the saddle-shaped trapezium. While various kinematic definitions have been used to describe the movements of the CMC-joint [7,8,9,10,11], in a majority of clinical applications, the major DOFs are defined as abduction/adduction and flexion/extension with coupled supination/pronation. The axes of rotation of CMC-joint abduction/abduction and flexion/extension are considered perpendicular, while the flexion/extension axes of all thumb joints are considered parallel with each other [8]. To define CMC-joint angles with this definition, the changing orientations of the abduction/adduction and flexion/extension axes with respect to the fixed reference frame on the hand needs to be considered when the thumb opposes. Thus, another DOF should be included that can track the variation in the orientation of the axes.

**Figure 1:**
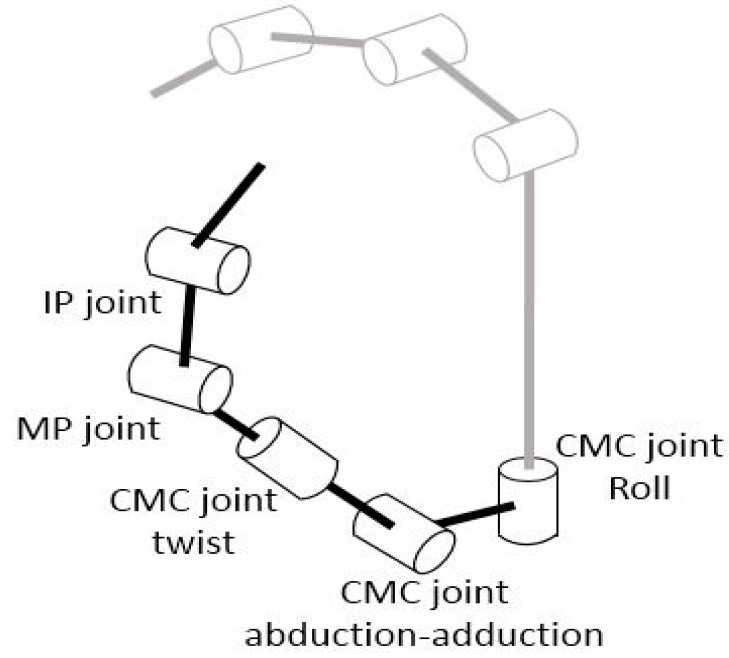
Kinematic model used in CyberGlove^®^.

The kinematic model of the CMC-joint used in the CyberGlove^®^ [11,16], which employs bending sensors, is shown in Figure 1. Unlike the clinical definition, the CyberGlove^®^ model uses CMC-joint roll motion instead of flexion/extension. The CMC-joint roll motion and twist motion (supination/pronation) in the model, however, are coupled to a certain degree, as they produce similar movements of the first metacarpal bone. The roll motion of the CMC-joint in this model does not represent the commonly used clinical definition of CMC-joint flexion/extension, as the axis of rotation is defined orthogonal to the second metacarpal bone, instead of the first metacarpal bone.

Therefore, in this study, we attempted to adopt the definition of the CMC-joint angle commonly used in clinical research [8], and we re-defined the CMC-joint roll motion such that it describes the varying orientation of the axis of rotation as the thumb opposes. The supination/pronation motion was excluded, as CMC-joint roll motion can describe this motion.

### 2.2. Proposed CMC-Joint Angle Definition

**Figure 2:**
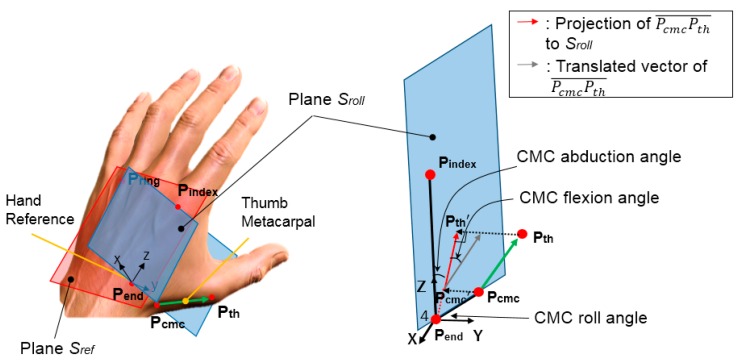
Kinematic definition of CMC-joint angles.

The kinematic definition of the CMC-joint proposed in this study is shown in Figure 2. Here, two planes and a vector corresponding to the thumb metacarpal bone were defined using five reference points (P_ring_: dorsal aspect of the ring finger metacarpophangeal (MCP) joint; P_index_: dorsal aspect of the index finger MCP joint; P_end_: end point of the second metacarpal bone that meets with the carpal bones; P_cmc_: dorsal aspect of the CMC-joint; P_th_: dorsal aspect of the thumb MCP joint). The hand reference plane, *S_ref_*, is defined by P_ring_, P_index_, and P_end_, while the second reference plane, *S_roll_*, is defined by P_index_, P_end_, and P_cmc_. The vector corresponding to the thumb metacarpal bone was defined as $\vec{P_{cmc}P_{th}}$ . The angle between *S_ref_* and *S_roll_* defines the CMC roll angle. The CMC flexion angle can then be defined as the angle between $\vec{P_{cmc}P_{th}}$ and *S_roll_*. Finally, the CMC abduction angle was defined as the angle between two vectors on plane *S_roll—_*$\vec{P_{cmc}{}^{\prime }P_{th}{}^{\prime }}$, the projected vector of $\vec{P_{cmc}P_{th}}$ on plane *S_roll_* and $\vec{P_{end}P_{index}}$. The proposed method to define three DOFs of the CMC-joint is different from previous biomechanical models [8] and/or kinematic models used in sensing devices [11,16], since the proposed model first computes the CMC roll angle as a basis to define the CMC-joint flexion/extension and adduction/abduction angles. The proposed definition not only allows external derivation of the CMC-joint flexion and abduction angles from the hand surface, but is also consistent with the clinical definition of the CMC-joint angles (*i.e.*, that the flexion/extension (abduction/adduction) axes of all thumb joints are parallel) [6].

## 3. Optimal Bending Sensor Locations for CMC-Joint Angle Measurement

### 3.1. Sensor Selection and Characteriztion

For compact design, a two-inch Bend Sensor^®^ (Flexpoint Sensor Systems Inc., Draper, UT, USA) was selected due to improved sensitivity and a smaller hysteresis effect than other similar available products [18]. First, to characterize the properties of this specific bending sensor, sensor output with varying bending angles and radii of curvature was examined. For a fixed radius of curvature, sensor output with respect to the bending angle could be modeled using two linear slopes [19]. For a fixed bending angle, sensor output decreased linearly with respect to the radius of curvature until the radius of curvature was larger than 60 mm (making the sensor almost flat), at which point there were no considerable changes in sensor output.

### 3.2. Method Overview

A brief overview of the techniques used to determine optimal sensor locations is provided in this section. The optimal positions and orientations of two CMC-joint-angle sensors (an abduction and a flexion sensor) were found by simulating bending sensor outputs at various candidate positions and orientations on the hand surface. Three-dimensional (3D) data points of the dorsal aspect of the hand surface near the CMC-joint were obtained from ten subjects using a 3D scanner. Then, sensor output was simulated using the radius of curvature and bending angles when the sensor was attached at a specific position with a specific orientation on the scanned hand surface. Sensor outputs at each candidate position and orientation were obtained for various CMC abduction and flexion angles. Then, the optimal sensor location, at which the sensitivity of the sensor with respect to the targeted DOF of the CMC-joint is maximized while the crosstalk from the other DOF is minimized, was found by examining the simulated sensor outputs.

### 3.3. Experimental Design

**Figure 3:**
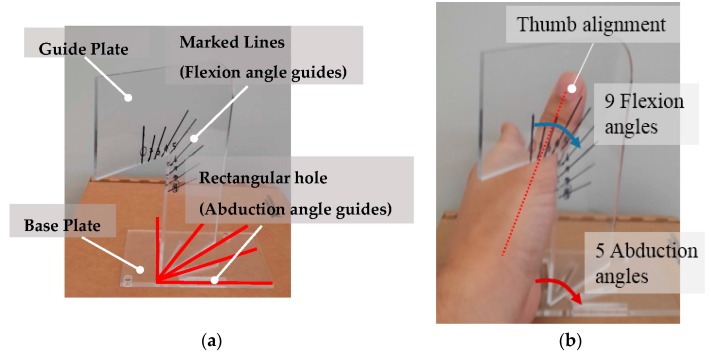
Thumb posture guide: (**a**) thumb posture guide overview; (**b**) thumb posing on the guide.

A 3D scanning system, SURFinder 3D sensor (CMES Inc., Seoul, Korea), was used for scanning the hand surface at various hand postures with *x*: 0.1 mm, *y*: 0.04 mm, *z*: 0.04 mm resolution. The dorsal surface of the hand was scanned for 45 different thumb postures—all possible combinations of five abduction angles and nine flexion angles of the CMC-joint. In order to standardize subject posing at the 45 thumb postures, combinations of various abduction angles and flexion angles, a custom thumb-posture guide was designed (Figure 3). The guide consists of two plastic plates, a base plate and a guide plate. The guide plate can be vertically placed on the base plate and the orientation of the guide plate can be adjusted using rectangular slots on the base plate. The base plate has five rectangular slots, which correspond to different CMC-joint abduction angles, spaced at 22.5-degree intervals. On the guide plate, nine lines are marked to indicate different CMC-joint flexion angles Figure 3a. The hand was placed on the base plate as shown in Figure 3b and the thumb was aligned flush against the marked lines on the guide plate. The subject was instructed to keep the hand still during scanning. For the subjects who had small hands, the subjects were not able to exactly align their thumbs with the marked lines for the lower flexion angles. In this case, the subjects were asked to keep their thumb parallel to the marked line instead of exact alignment. With this condition, the posture guide was applicable for all the subjects despite the different size of their hands, as the purpose of the guide was to variate 45 distinguishing thumb postures within a subject.

The experimental protocol was approved by the Institutional Review Board at the Korea Advanced Institute of Science and Technology (KH2015-10). Five male subjects (age range: 23–27 years) and five female subjects (age range: 22–52 years) provided informed consent and participated in the experiment.

### 3.4. Analysis

Finding the optimal CMC-joint-angle sensor locations from the 3D hand surface data required three steps. First, the 3D-point-cloud data were conditioned. Second, the radius of curvature and bending angle of the sensor were computed for various positions of the sensor origin and orientations of the sensor on the hand surface. The sensor outputs were simulated from this information. Lastly, the optimal positions and orientations of the CMC-joint-angle sensors, at which the sensors has greater sensitivity to the targeted DOF and minimal crosstalk due to the other DOF, were determined.

#### 3.4.1. Data Conditioning

**Figure 4:**
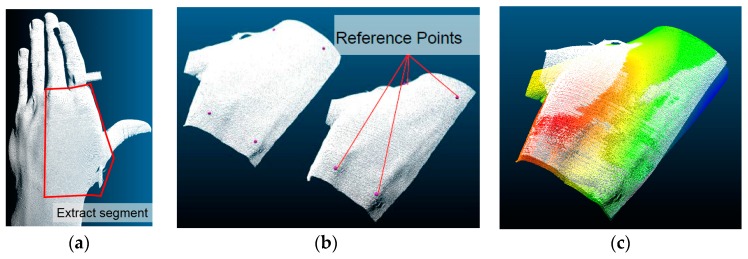
3D hand surface data conditioning (Subject 3): (**a**) cropping out unused data points; (**b**) align (point pairs picking) process; (**c**) alignment image after fine alignment process.

As we focused on the deformation of the hand surface by the CMC-joint movements, only the hand surface area containing the first, second, and third metacarpal bones was considered (Figure 4a). Other areas of the hand were cropped from the point-cloud data using the CloudCompare open source software (CloudCompare, (version 2.6.1) (GPL software) (2015)) [20] segmentation function. A representative image of the 3D point cloud after the cropping process is shown in Figure 4b.

After data cropping, the 45 hand surface 3D clouds obtained from each subject were aligned to a reference hand surface. The initial alignment between the two data clouds was performed by selecting four common reference points among the two data clouds (Figure 4b). The ‘align (point pairs picking)’ function of CloudCompare was used for this alignment. After the four-point-picking alignment, fine adjustment was conducted using the ‘fine adjustments’ function that uses the Iterative Closest Point (ICP) method. A representative alignment result is shown in Figure 4c.

**Figure 5:**
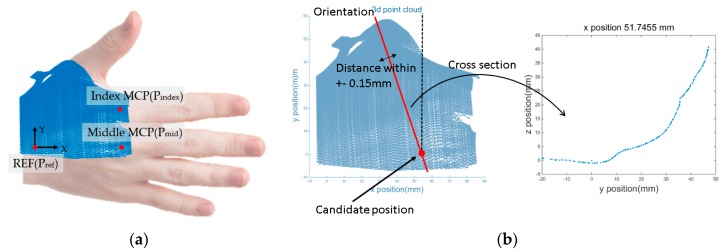
Coordinate transformation and curve derivation along the hand surface at the candidate location: (**a**) transformed image of 3D hand surface points with respect to the reference frame; (**b**) derivation schematic of the curve along the hand surface.

After the alignment, the 3D hand surface points were transformed to a reference frame coordinate. The *x*-axis was defined as the distal direction of the middle finger metacarpal bone. The *z*-axis was defined as the normal vector of the plane made by points on the index finger MCP joint (P_index_), middle finger MCP joint (P_mid_), the end of the middle finger metacarpal bone that meets with the wrist (P_ref_) in the palmer direction. The *y*-axis was defined as normal to both the *x*- and *z*-axes. The reference frame was positioned on P_ref_ as shown in Figure 5a.

A rotational matrix, R, and the transformation matrix, T, were defined with the unit reference *x-*, *y-*, *z*-axis vectors using the following equations:

(1) $$R={\left[\begin{array}{ccc} x & y & z \end{array}\right]}^{-1}$$

(2) $$T=\left[\begin{array}{cc} R & -R\cdot p\\ 0 & 1 \end{array}\right]$$

where *x*: *x*-axis unit vector, *y*: *y*-axis unit vector, *z*: *z*-axis unit vector, p: P_ref_ coordinate.

The transformation matrix, T, was applied to the coordinates of each 3D point of the hand surface (*p* = [*x*; *y*; *z*; 1]). All computations were conducted using a custom MATLAB program (MathWorks Inc., Natick, MA, USA).

After the transformation, the curve along the hand surface was derived for 44 candidate positions (11 *x*-coordinates and 4 *y*-coordinates; each coordinate having the same interval) and 36 orientations. The candidate orientations were set at −90° to 90° in five-degree increments. For a single candidate location, an intersection plane, *S_int_*, was defined that intersects with the candidate position, is directed along the candidate orientation, and is placed normal to the *x*–*y* plane. The 3D hand-surface data points intersecting with *S_int_* were defined as the points that were within 0.15 mm from *S_int_*. An illustration of deriving the curve along the hand surface is shown in Figure 5b.

#### 3.4.2. Estimation of Sensor Outputs

The degree of deformation of the bending sensor was computed from the hand surface data and the sensor outputs were simulated from the deformed shape. From the estimated shape of the sensor, the sensor output was estimated by deriving the radius of curvature and the bending angle of the sensor. In order to derive the radius of curvature and bending angle of the sensor placed on a certain location, the curve along the hand surface exceeding the length of the two-inch Bend Sensor^®^ was trimmed starting at the candidate position. Then, a circle was fit to the trimmed curve (circlefit function; MATLAB). The radius of the best-fit circle was defined as the radius of curvature of the sensor. The first point and end point of the trimmed curve was connected to the center of the best-fit circle, and the angle made by these lines was considered the bending angle of the sensor. When the radius of the best-fit circle exceeded a preset threshold (60 mm), sensor output was considered to be 0 V.

Through testing of the two-inch Bend Sensor^®^, the sensor output with respect to the bending angle and the radius of curvature was modeled using Equation (3). The sensor output has a linear relationship with the bending angle and radius of curvature. The contribution of the radius of curvature to the sensor output is negligible when the radius of curvature exceeds 60 mm.

(3) $$V=\{\begin{array}{c} 0.01916\theta -0.07r\;(r\leq 60)\\ 0.01916\theta -2.07\;(r>60) \end{array}$$

where *V*: Estimated sensor output, θ: Bending angle, and *r*: Radius of curvature.

#### 3.4.3. Optimizing Sensor Locations

For each candidate location on the hand surface, 45 sensor outputs were estimated, as there were 45 sets of 3D hand surface data with different thumb postures. The 45 sensor outputs were plotted with respect to the CMC-joint flexion and abduction angle, derived from the definition introduced in Section 2.

Least squares linear regression to determine the slope of the curve was performed to correlate sensor outputs with CMC-joint angles (abduction or flexion angle), and the magnitude of the slope was determined. The magnitude of the slope represents the sensitivity of the sensor with respect to the corresponding CMC-joint angle. In order to find the optimal location of the CMC-joint angle sensor, the sensitivity to the targeted CMC-joint movement (abduction or flexion) should be maximized while the sensitivity to the other CMC-joint movement (flexion or abduction) should be minimized. The normalized root mean square error (RMSE) of the linear regression for the sensor output with respect to the targeted movement was also determined. Minimization of the normalized RMSE, minimizes crosstalk. The normalization was performed with respect to the variation of the sensor output and the CMC-joint angle. The magnitude of the slope and the normalized RMSE of the linear regression model were used as parameters for the cost functions shown in Equations (4) and (5):

(4) $$J_{ab}\left(x,y,\phi \right)=w_{1}\cdot \left(\frac{Var_{ab}}{a^{2}{}_{ab}(x,y,\phi )}\right)+w_{2}\cdot \left(\frac{a^{2}{}_{flex}(x,y,\phi )}{Var_{flex}}\right)+w_{3}\cdot (e^{2}{}_{ab}(x,y,\phi ))$$

(5) $$J_{flex}\left(x,y,\phi \right)=w_{1}\cdot \left(\frac{Var_{flex}}{a^{2}{}_{flex}(x,y,\phi )}\right)+w_{2}\cdot \left(\frac{a^{2}{}_{ab}(x,y,\phi )}{Var_{ab}}\right)+w_{3}\cdot (e^{2}{}_{flex}(x,y,\phi ))$$

$J_{ab,flex}(x,y,\phi )$ is cost function of abduction and flexion on position (*x*, *y*) with ϕ orientation. $w_{i}$ is weighting; maximizing sensor sensitivity to target movement is *i* = 1; minimizing sensor sensitivity to other movement is *i* = 2; minimizing normalized RMSE is *i* = 3. $a_{ab,flex}$ is linear fitting slope magnitude with respect to CMC-joint abduction and flexion angle. $e_{ab,flex}$ is normalized RMSE error of linear fitting of sensor signal with respect to CMC-joint abduction and flexion angle. $Var_{ab,flex}$ is variance of $a_{ab,flex}$ for every (x,y,ϕ) coordinate.

**Figure 6:**
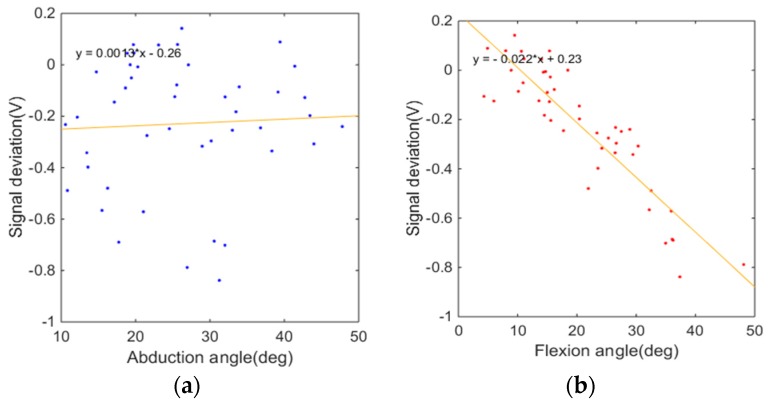
Representative CMC-joint flexion sensor response with the sensor in the optimal location. (x,y,ϕ)=(0.5,0.333,−39°) for Subject 3: (**a**) sensor response with respect to CMC-joint abduction angle; (**b**) sensor response with respect to CMC-joint flexion angle.

The optimal sensor locations of the CMC-joint abduction sensor and CMC-joint flexion sensor were derived from the location that minimized the cost functions, *J_ab_* and *J_flex_*, at which the sensitivity to the targeted CMC-joint movement is maximized while the crosstalk with respect to the other CMC-joint movement is minimized. The weightings were set as *w*_1_ = 20, *w*_2_ = 1, and *w*_3_ = 5, as the most important factor in the cost function was to maximize the sensor sensitivity with respect to the targeted movement. The representative sensor response result with the CMC-joint-angle sensor placed at the optimal location is illustrated in Figure 6 (optimally placed flexion sensor). It is noted that the sensor output shows a clear linear decrement with respect to the flexion angle, while it does not show a clear linear trend with respect to the abduction angle. This illustrates a high sensitivity for the targeted movement (flexion) with a low sensitivity for abduction.

**Figure 7:**
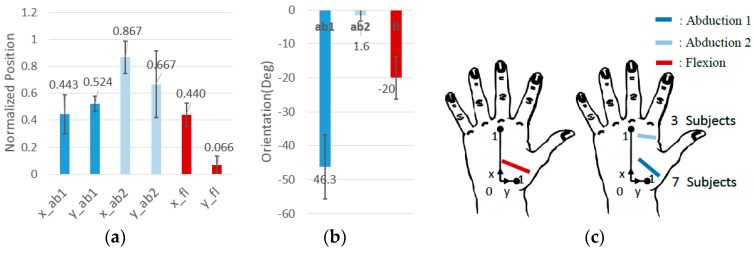
Average optimal CMC sensor position and orientations (10 subjects): (**a**) average optimal CMC-joint angle sensor positions; (**b**) average optimal CMC-joint angle sensor orientations; (**c**) illustration of the optimal CMC-joint angle sensor location. The abduction sensor had two distinct location trends. The optimal sensor location of seven subjects was across the adductor pollicis (mean: *x* = 0.442, *y* = 0.524, orientation = −46.3°), and the optimal sensor location for three subjects was across the 2nd metacarpal bone (mean: *x* = 0.867, *y* = 0.667, orientation = −1.7°).

The optimal locations of the CMC-joint angle sensors (abduction and flexion sensors) were determined for ten subjects, and the average locations are illustrated in Figure 7. The position was normalized with respect to the size of the subject’s hand measured from the collected 3D point cloud of the hand surface. The *x* position and *y* position was normalized by the length of $\vec{P_{ref}P_{mid}}$ and $\vec{P_{mid}P_{index}}$, respectively (Figure 5a) that were measured with length measurement tool of the CloudCompare software. The flexion sensor originated from the lower half of the dorsal hand near the 3rd metacarpal and pointed toward the first metacarpal near the CMC-joint. The abduction sensor showed a large variance in optimal orientation due to two distinguishing trends in abduction sensor optimal location. The sensors both originated from the upper half of the dorsal hand between the 2nd and 3rd metacarpals, while one sensor was placed across the adductor pollicis, the other sensor was placed across the 2nd metacarpal bone (Figure 7). In the former orientation, the sensor output increases as the CMC-joint adducts, while it decreases for the latter orientation. In the former orientation, the sensor output increases as the CMC-joint adducts, while it decreases for the latter orientation. The two distinguishing trends are expected to be related to the thickness of the adductor pollicis muscle. For subjects having thicker adductor pollicis, the surface near the muscle bulges when it contracts (or CMC-joint adduction) causing the sensor placed across the muscle to bend and the sensor output to increase. However, for subjects with thin muscle, the sensor output is dominated by bone movement rather than the shape of the muscle. For subjects with thin adductor polices muscle, placing sensors at the latter location obtains better sensor outputs since one end of the sensor follows the movement of the hand web (skin between the 1st and 2nd metacarpal), which rotates around the 2nd metacarpal bone during the ab/adduction motion. The sensor becomes flat when the CMC-joint adducts (decreasing sensor output), while it bends as the CMC-joint abducts (increasing sensor output).

#### 3.4.4. Crosstalk Analysis for Optimally-Located Sensors

**Figure 8:**
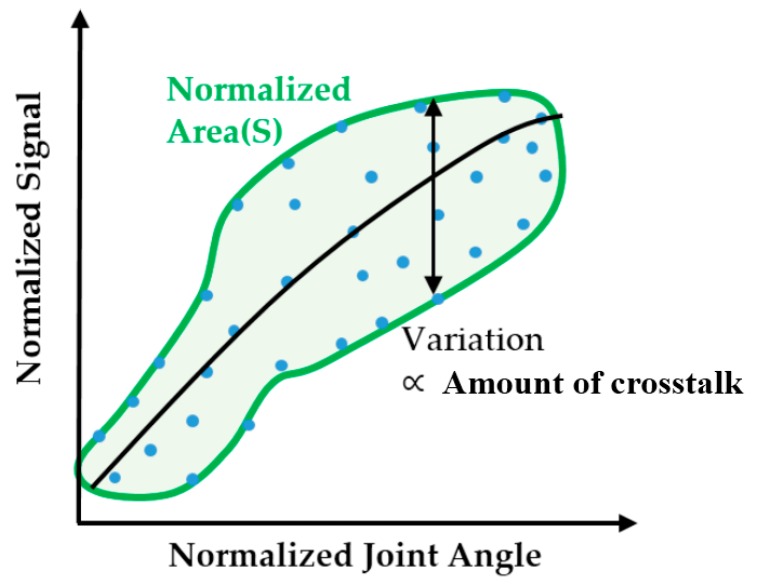
Deriving the magnitude of crosstalk of the CMC-joint angle sensor from a plot of the normalized sensor output with respect to normalized joint angle. The magnitude of the normalized area, S, represents the overall magnitude of crosstalk.

In order to ensure the validity of optimal sensor locations as determined using the methods described above, the magnitude of sensor crosstalk was first simulated and compared with experimental results. The magnitude of crosstalk was calculated from the simulated sensor output with respect to the CMC-joint angles (abduction and flexion angle) derived from the 3D hand surface data when the sensor is placed in the optimal location. Representative plots are shown in Figure 6. The magnitude of crosstalk was defined as the normalized area calculated from the contour of the plot of estimated sensor output *versus* target joint angle. A conceptual graph (Figure 8) illustrates the definition of crosstalk magnitude.

In an ideal case, where there is no variability in sensor output at a fixed CMC-joint angle combination (flexion and abduction angle), the signal of the sensor for the measurement of the targeted CMC-joint angle (flexion or abduction angle) will be only disturbed and variate by the other CMC-joint angle (abduction or flexion angle) movement. It is important to have a smaller normalized area to minimize crosstalk. If the sensor does not have any crosstalk with the other joint motion (*i.e*., normalized area = 0), the sensor output will map to the target joint angle following a certain function (illustrated with the black line in Figure 8). However, if the sensor is affected by other joint motions, sensor output will vary at each target joint angle and the magnitude of the variation will enlarge with larger magnitudes of crosstalk (*i.e.*, increased normalized area). Therefore, minimizing the normalized area is important to achieve a truly decoupled measurement of CMC-joint angle.

The magnitude of crosstalk in sensor output was quantified by examining the normalized area in the plot described above. The threshold was set to 0.5, which indicates that approximately 50% of the variability in sensor output is due to confounding effects from undesired motions. If the targeted movement explains more than half of the variability in sensor output, the normalized area will be less than 0.5. Of note, the normalized area (variability) also includes noise present in sensor output and noise present in the surface contour obtained from 3D scanning. However, the effects of such noise/uncertainty on the overall variability of the sensor output are much smaller than those from potential crosstalk. For example, the standard deviation of CMC-joint angle from surface data derived from a single hand was 2.2°. If we assume that the degree of variation remains similar across all postures, the normalized area due to measurement noise would be 0.136, much smaller than the majority of normalized areas from the simulation results (~0.7). Given the relative magnitudes of these sources of variability, we can consider crosstalk from the untargeted sensor outputs the primary source of variability that accounts for the normalized area.

The normalized areas (magnitude of crosstalk) of the optimally-located CMC-joint abduction and flexion sensor for each subject are shown in Table 1. The average normalized area was 0.298 for the flexion sensor and 0.275 for the abduction sensor. It is noted that the targeted movement always had greater effect on sensor output, as the magnitude of crosstalk was less than 0.5 for all subjects. Excluding the effect of variability in the data itself (≈0.14), which is included in the normalized area, approximately 15% of the variability of the sensor output was due to undesired movements. Considering that CMC-joint sensor output variation due to undesired movements ranged from 12.6% to 79.2% in a previous study, in which crosstalk was briefly assessed by placing a bending sensor on several locations on the hand surface [19], the degree of crosstalk obtained in the current study can be considered minimal.

## 4. Experimental Evaluation

### 4.1. Experimental Protocol

**Figure 9:**
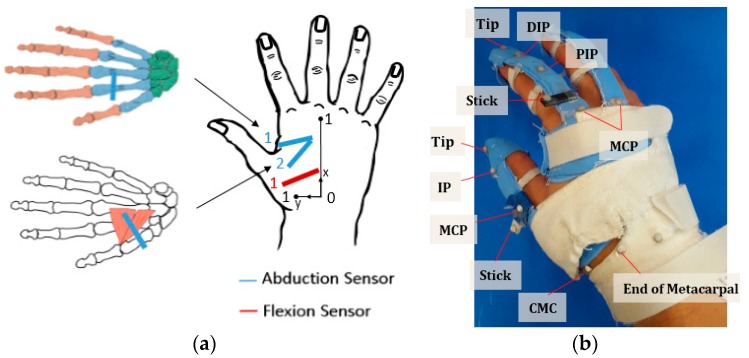
Sensor and marker positions for the experiment: (**a**) sensor locations; (**b**) marker placement for CMC-joint-angle estimation.

The simulated optimal sensor locations were evaluated by placing the bending sensors on the derived optimal locations and observing the sensor response with respect to the CMC-joint angle. For stable sensor readings, it was important to ensure that the bending sensor closely adhered to the hand surface. Therefore, it was embedded between two flexible fabric straps made of Lycra^®^ (Wichita, KS). The flexible fabric strap was partially (either one or two ends of the strap) sewed on a glove structure, in order to fix the sensor at the optimal position, while reducing the strain effect delivered through the glove structure. The flexion sensor was placed at the single optimal location, in which the sensor pointed toward the CMC-joint and originated from the midpoint of the third metacarpal. The abduction sensor was placed at two optimal locations: Location 1 across the index metacarpal and Location 2 across the adductor pollicis (Figure 9a).

After placing the sensors, reflective markers were placed on the hand as shown in Figure 9b. The CMC abduction and flexion angles were calculated post hoc from the marker positions, using the kinematic definitions described in Section 2. With the markers and bending sensors attached to the hand surface, the subject conducted a circumduction motion (*i.e.*, rotating the thumb along the largest circle he or she could make). The circumduction motion was selected in order to collect various CMC-joint abduction and flexion angle combinations. The marker positions and sensor outputs were collected using a Vicon motion capture system (Vicon Motion Systems Ltd., Oxford, UK) consisting of eight T-series cameras and Giganet DAQ box (Vicon Motion Systems Ltd., Oxford, UK ). The CMC-joint angles were calculated using a custom MATLAB program. Four male subjects (age range: 22–35 years) consented and participated in the experiment. None of them were chosen from the pool of the ten subjects who participated in the experiment in the previous section because we wanted to check how the optimal sensor locations apply to the other subjects. Obviously, the sensing error can be minimized by customizing sensor locations for individual users; however, individual customization might not be practical in typical applications. It might be reasonable to assume that small-, medium-, and large-size glove systems would be practical in final product design. For the evaluation experiment, we prototyped a medium size sensing system (width: 80–90 mm) and recruited subjects who fit in the range of medium size.

### 4.2. Results

#### 4.2.1. Crosstalk Magnitude

**Figure 10:**
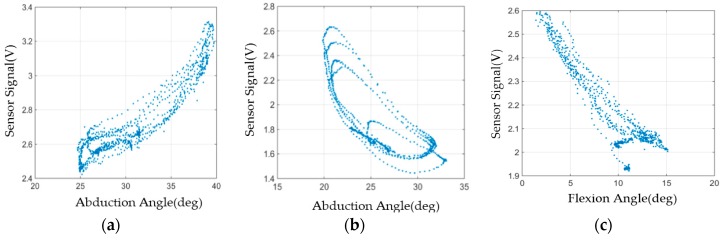
Sensor response with respect to the targeted CMC-joint angle with sensors located in the optimal positions during circumduction motion (rotation of the thumb along a circle): (**a**) CMC abduction sensor response with sensor in Location 1 (across second metacarpal); (**b**) CMC abduction sensor response with sensor in Location 2 (across adductor pollicis); (**c**) CMC flexion sensor response.

Representative results of the sensor output with respect to the targeted CMC-joint angle (abduction and flexion angle) are shown in Figure 10.

Abduction sensor results suggest that the optimal abduction sensor location (with the smallest magnitude of crosstalk) was either one of the two previously determined optimal locations that are illustrated in blue lines in Figure 9a. The flexion sensor showed smaller variation with respect to the other movements compared to the abduction sensor. Table 2 shows the normalized area (crosstalk magnitude) of the sensors, calculated from the contour of the plot of sensor output *vs.* target joint angle for each subject. The average normalized area of the abduction sensor, in the location with lower crosstalk, was 0.281, while the average normalized area for the flexion sensor was 0.279. Sensor hysteresis was quantified as 0.099 by testing the bending sensor through multiple bend-and-extend cycles. Discounting the hysteresis effects, the measured magnitude of crosstalk was similar to the simulated values, suggesting a high validity of the simulated results.

#### 4.2.2. Accuracy of CMC-Joint Angle Estimation

**Figure 11:**
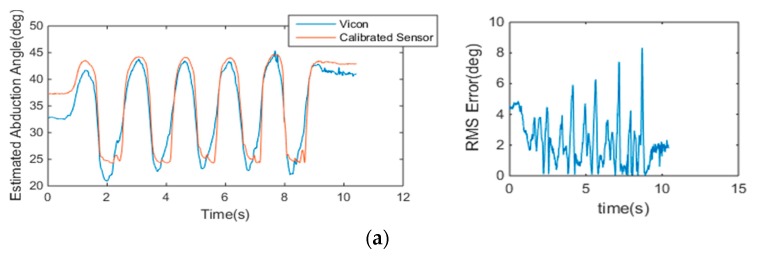

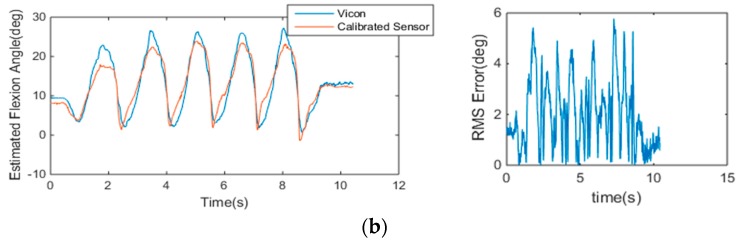
Representative results for response of the calibrated sensor with respect to the CMC-joint angles: (**a**) CMC-joint abduction angle estimated with Vicon and with the calibrated abduction sensor (**left**) and the RMSE of the calibrated abduction sensor with respect to the Vicon results (**right**); (**b**) CMC-joint flexion angle estimated with Vicon and with the calibrated abduction sensor (**left**) and the RMSE of the calibrated flexion sensor respect to the Vicon results (**right**).

As this study focuses on the improvements made by placing the sensors in optimal locations, sensor calibration was performed in a simple manner. The calibration for the CMC sensors was conducted using the relationship between CMC-joint angles and sensor outputs (Representative plots are shown in Figure 10). The sensor outputs were transformed to CMC-joint angles (flexion and abduction angles). The calibration was done using the polyfit function in MATLAB. For the abduction sensor, the sensor location that gave a lower magnitude of crosstalk was calibrated. The CMC-joint angles that were derived from marker positions collected with the Vicon system were used as the gold standard reference. Representative results after the calibration are shown in Figure 11. The considerably larger RMSE near the minimum and maximum angles originated from the hysteresis effect of the sensor and shows a different sensor response depending on the phase (*i.e.*, whether it is in the increasing phase or the decreasing phase). The accuracy of the estimations is expected to improve by compensating for the hysteresis effect.

The RMSE of the sensor calibration results with respect to the reference joint angle are shown in Table 3 for each subject. The average RMSE of the flexion sensor was 2.8° and the average RMSE of the abduction sensor was 1.9°. It is noted from the results that the overall RMSEs were smaller than those presented in previous studies for other data gloves [15,16].

## 5. Discussion

While challenging to accurately measure using compact equipment, measurement of the thumb CMC-joint angle is important when estimating the thumb configuration in hand rehabilitation applications and in clinical assessment. In this study, the optimal locations of bending sensors (two-inch Bend Sensor^®^) for CMC-joint measurement were determined using a novel systematic method that employed a 3D scanning technique. Our optimization-based simulation resulted in similar sensor locations for most subjects, while the specific position and orientation varied slightly across subjects. The optimally located flexion sensor originates from the middle of the dorsal hand and is oriented toward the CMC-joint. Interestingly, the optimal location/orientation of the abduction sensor showed two distinct trends. One was similar to that of the adductor pollicis muscle, while the other was placed across the second metacarpal. When the sensors were optimally placed, the magnitude of crosstalk with movements other than the targeted DOF was minimized, such that the movement of the targeted joint angle was the dominant effect on sensor output.

Optimal positioning of the CMC-joint angle sensors also improved the accuracy of joint angle estimation. By placing the bending sensors in the optimal locations and using the clinical definitions of the DOFs of the CMC-joint movement, the accuracy of the CMC-joint angle estimation was improved. After simple calibration, even without compensating for crosstalk, the CMC-joint angle measurement error was improved to 2.8° ± 1.9° for the flexion sensor and 1.9° ± 1.2° for the abduction sensor-lower than the error values reported in previous studies [15,16]. However, direct comparison with these previous studies may be difficult due to the difference in the kinematic definition of the CMC-joint angles.

## 6. Conclusions

In this study, we introduced a methodology to find the optimal sensor locations for accurate measurement of the CMC-joint angle. On the optimal locations, the sensor had minimal crosstalk with the other thumb movements, undesired to be measured. The accuracy of the CMC-joint measurement has improved by placing the sensors on the derived location. However, it should be acknowledged that, while the crosstalk was reduced by adjusting the sensor locations, it was not completely eliminated. As the maximum estimated error exceeded the required accuracy for typical clinical assessment (5°) [17], additional improvements in sensor positioning and data processing may further reduce crosstalk magnitude, which, in turn, will improve the accuracy of joint angle estimation. For example, we could place the sensors on the individually specified optimal locations instead of the generalized locations to improve the accuracy of the estimation. In addition, further reduction of the crosstalk magnitude may be made by developing a calibration method that utilize the correlation between the sensor outputs of the optimally located abduction sensor and flexion sensor. The correlation would be used to filter out the crosstalk remaining in joint angle sensors.

Although the optimal sensor locations were found for specific bending sensors (Bend Sensor^®^), the proposed methodology is general and could be extended to any other type of flexible sensors by considering sensor dynamics. Furthermore, the results of this study could be employed in the design of wearable robots for the hand, for which the use of an estimated thumb posture as a feedback output would be critical for the accurate control of motion.

In conclusion, the accurate hand sensing system enables more precise hand assessment as well as functional training of skillful hand tasks specifically during rehabilitation procedures. It can be applied to general training systems such as a virtual-reality based game applications. Moreover, the methodology provided in this study can be applied to enhance accuracy of other wearable sensing systems.

## Figures and Tables

**Table 1:** Normalized areas for each subject when CMC-joint angle sensors are placed at optimal locations.

Subject	S1	S2	S3	S4	S5	S6	S7	S8	S9	S10	Mean
**Normalized area (flexion sensor)**	0.384	0.234	0.190	0.349	0.154	0.316	0.307	0.315	0.176	0.324	0.275
**Normalized area (abduction sensor)**	0.255	0.275	0.239	0.367	0.372	0.308	0.341	0.353	0.203	0.298	0.298

**Table 2:** Normalized area at the optimal sensor locations.

Subject	Normalized Area		
	Abduction Sensor 1	Abduction Sensor 2	Flexion Sensor
S1	0.272	***0.210***	0.278
S2	0.462	***0.352***	0.334
S3	***0.278***	0.461	0.242
S4	0.644	***0.285***	0.260
Avg.	0.281 ± 0.0581		0.279 ± 0.0399

Bold numbers represent abduction sensor location with smaller normalized area; the average (avg.) normalized area for the abduction sensor is calculated from bold values.

**Table 3:** The normalized root mean square error (RMSE) of calibrated sensor values.

Subject	RMSE (°)	
	Abduction Sensor	Flexion Sensor
S1	1.6 ± 1.1	1.6 ± 0.96
S2	2.6 ± 1.1	4.6 ± 1.7
S3	1.3 ± 0.94	3.1 ± 1.4
S4	2.2 ± 1.2	2.0 ± 1.2
Avg.	1.9 ± 1.2	2.8 ± 1.9
